# Oral paracoccidioidomycosis in a non-endemic region from Brazil: A short case series

**DOI:** 10.4317/jced.56199

**Published:** 2019-10-01

**Authors:** Reydson-Alcides-de Lima Souza, Paulo-Rogério-Ferreti Bonan, Mariana-Bitu-Ramos Pinto, José-Divaldo Prado, Jurema-Freire-Lisboa de Castro, Elaine-Judite-de Amorim Carvalho, Danyel-Elias-da Cruz Perez

**Affiliations:** 1 DDS, MSc student, Piracicaba Dental School, Oral Pathology Area, State University of Campinas, Piracicaba, São Paulo, Brazil; 2 DDS, PhD, School of Dentistry, Stomatology Unit, Universidade Federal da Paraíba, João Pessoa, Paraíba, Brazil; 3 DDS, A. C. Camargo Cancer Center, Department of Stomatology, São Paulo, São Paulo, Brazil; 4 DDS, PhD, Professor, School of Dentistry, Oral Pathology Unit, Universidade Federal de Pernambuco, Recife, Pernambuco, Brazil

## Abstract

**Background:**

Although the paracoccidioidomycosis (PCM) is endemic in Brazil, the occurrence in most states from the North and Northeastern Brazil is very unusual. The aim of this study was to evaluate the clinicopathologic features of a case series of oral PCM in a non-endemic region from Brazil (Northeastern region), discussing the clinical and histopathological differential diagnoses of the oral manifestations of the disease.

**Material and Methods:**

Between 2000 and 2017, all cases of oral PCM were retrieved from the Oral Pathology Laboratory, Universidade Federal de Pernambuco, located at Northeastern Brazil. Clinical data, such as age, gender, origin, occupation, site, symptoms, time of complaints, clinical presentation, number of lesions, and clinical hypotheses of diagnosis, were collected from the clinical charts. All cases were histologically reviewed in hematoxylin-eosin and Gomori-Grocott staining.

**Results:**

Six cases were identified. All patients were male, with a mean age of 53.8 years (ranging from 40 to 73 years). Four cases appeared as multiple ulcers and two presented single lesions (buccal mucosa and hard palate). Clinically, in five cases, squamous cell carcinoma was considered in the differential diagnosis. The common histopathological features consisted of hyperplastic epithelium, intraepithelial microabscesses, and formation of granulomatous chronic inflammatory reaction in a fibrous connective tissue with severe chronic inflammatory reaction. Yeasts were observed either inside of multinucleated giant cells or extracellularly.

**Conclusions:**

Although rare in non-endemic regions, oral PCM should be considered in the differential diagnosis of oral chronic ulcers, mainly those multiple.

** Key words:**Oral mucosa, mycology, paracoccidoidomycosis, ulcer.

## Introduction

The paracoccidioidomycosis (PCM) is a systemic mycosis caused by the dimorphic fungi *Paracoccidioides brasiliensis*. It represents a significant infection in South America, occurring mostly in tropical and subtropical countries, such as Brazil, Venezuela, Argentina, and Colombia (1). Brazil is considered an endemic country, with a higher prevalence of PCM in the South, Southeast, and Midwest regions (2). Other highly endemic areas include the States of Para, Rondonia, Tocantins, and Maranhao (3).

PCM has a strong predilection for middle-aged men, usually habitants of rural areas. It is acquired by inhalation of spores, primarily affecting the lungs. The infection may spread by hematogenous or lymphatic route, affecting other organs and regions, such as the skin and oral mucosa. Oral manifestations help in the clinical diagnosis of PCM and are often the first manifestations perceived by patients. The oral lesions usually appear as multiple shallow and painful ulcers of moriform aspect (classic appearance of strawberry skin) with irregular contour, reaching mainly the alveolar mucosa, gingiva, and palate (4). Difficulty in chewing and bad breath are other reported symptoms. The PCM may present systemic signs and symptoms, such as fever, cough, fatigue, lymphadenopathy, and weight loss (5,6).

Although the PCM is endemic in Brazil, the occurrence in most states from the North and Northeastern Brazil is very unusual. Recently, from 320 cases of oral PCM in a large multicentre study in Brazil, only one case (0.3%) occurred in Northeastern region (7). Thus, this study aimed to evaluate the clinicopathologic features of a case series of oral PCM in a non-endemic region of Brazil, discussing the clinical and histopathological differential diagnosis of the oral manifestations of this disease.

## Material and Methods

This retrospective, observational, descriptive study was approved by the Institutional Review Board (protocol number: 44536715.8.0000.5208). Between 2000 and 2017, all cases of oral PCM diagnosed in the Oral Pathology Laboratory of the Universidade Federal de Pernambuco, Recife, Pernambuco, Brazil, were selected for this study. Clinical data, such as age, sex, origin, occupation, site, symptoms, time of complaints, clinical presentation, number of lesions, and clinical hypothesis of diagnosis were collected from the clinical charts.

Histopathological slides stained with hematoxylin and eosin, periodic-acid Schiff (PAS), and Grocott-Gomori (silver impregnation) were reviewed to confirm the diagnosis. After data collection, they were tabulated and analyzed by descriptive statistics using the Statistical Package for Social Sciences (SPSS) program, version 20, with relative and absolute distribution of clinical and histopathological data.

## Results

**Figure 1:**
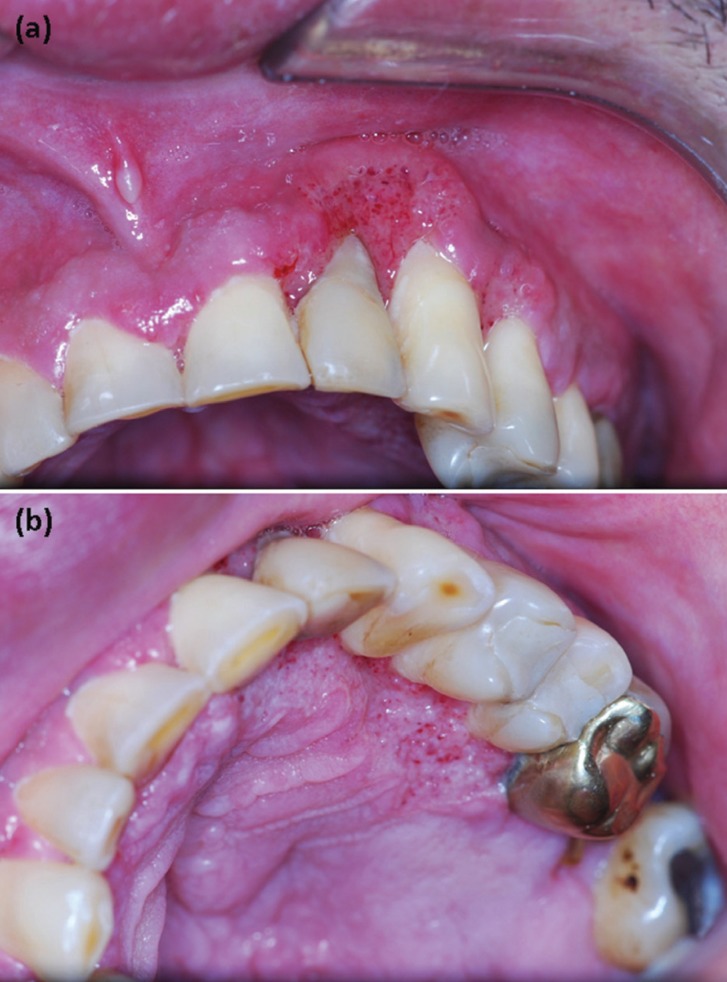
A) Erythematous and irregular ulcer, with granular surface, located in the buccal gingiva. B) Moriform ulcer in palatine gingiva.

Among the 6200 cases of oral lesions diagnosed within the study period, 6 (0.09%) were of oral manifestations of PCM ( Table 1). All cases were of male patients, with a mean age of 53.8 years (range, 40–73 years). Five cases (83.3%) occurred in patients living in the Northeast region and 1 (16.7%) from the Southeastern Brazil. Two (33.3%) patients were retired rural workers, 1 farmer (16.7%), and 1 upholsterer (16.7%). In two cases (33.3%), the occupation was not identified. The mean time of complaint was 60 days (range, 20–120 days). Multiple oral lesions appeared in four cases (66.7%) and a single oral lesion in two cases (33.3%). As regards symptoms, all patients complained of pain. The most common clinical features were irregular ulcers, with erythematous and granular surface, and superficial bleeding points (moriform aspect) (Fig. 1). The most frequently affected site was the buccal mucosa, followed by soft palate, hard palate, and gingiva. Squamous cell carcinoma was considered the clinical hypothesis of diagnosis in five cases and PCM in four cases. Other clinical diagnoses considered were leishmaniasis, histoplasmosis, and actinic cheilitis ( Table 1).

**Table 1:**
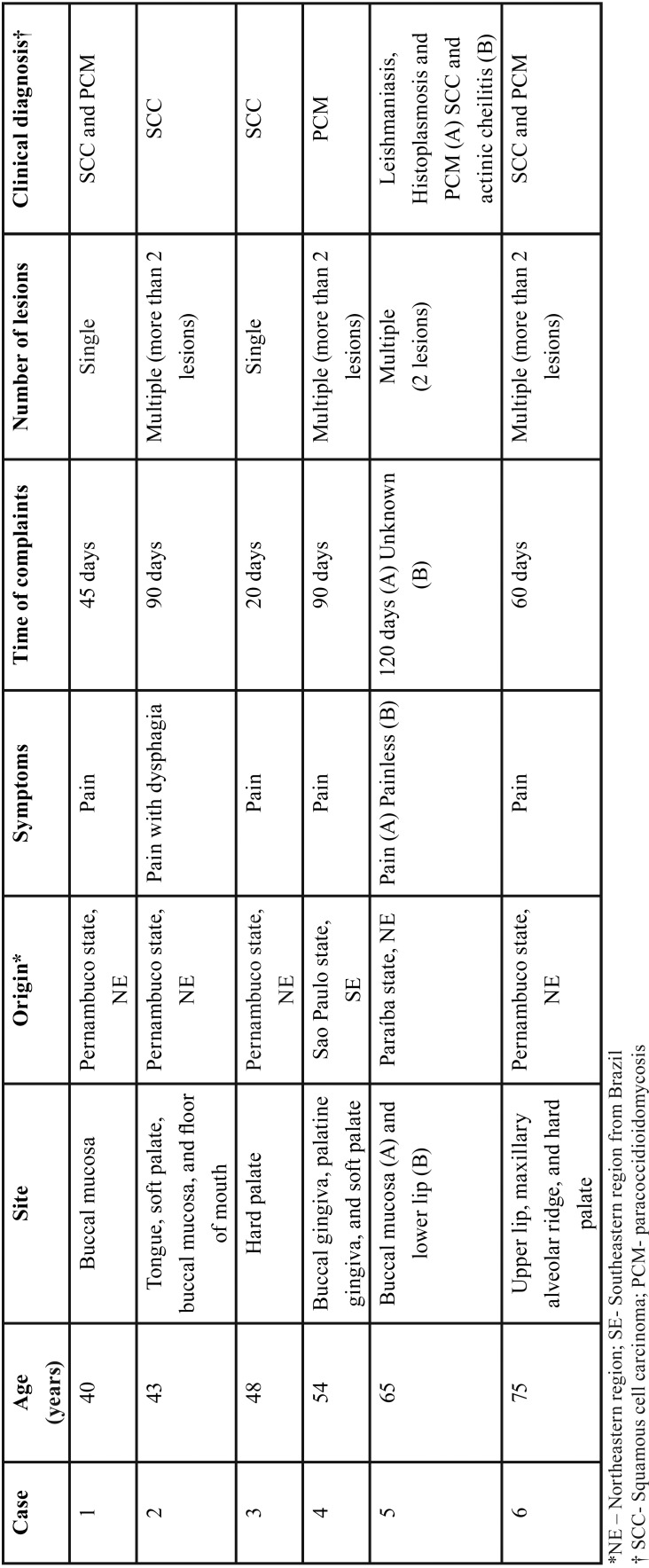
Clinical data of 6 cases of oral paracoccidoidomycosis in a non-endemic region from Brazil.

**Figure 2:**
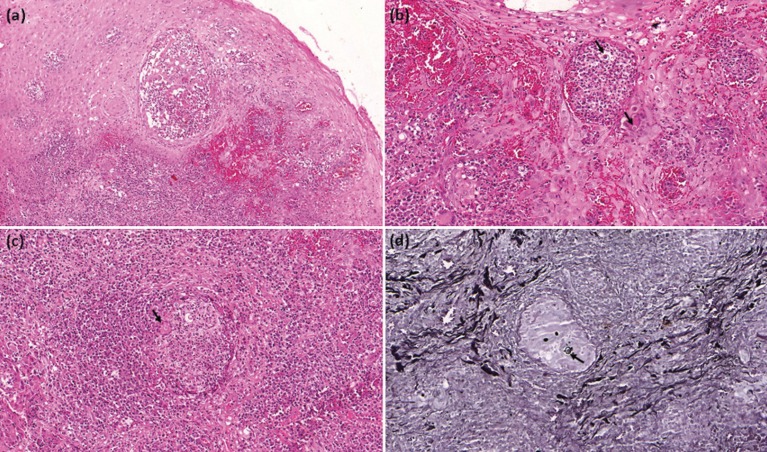
A) Epithelial hyperplasia with presence of microabscess (hematoxylin-eosin, original magnification, x100). B) Intraepithelial microabscess with presence of extracellular yeasts and inside of multinucleated giant cell (arrows) (hematoxylin-eosin, original magnification, x200). C) Severe chronic inflammatory reaction with formation of granuloma. Multinucleated giant cells are observed (arrow) (hematoxylin-eosin, original magnification, x200). D) Yeasts showing globous aspect, with multiple buddings of mickey ear appearance (Gomori-Grocott, original magnification, x200).

Microscopically, all cases presented acanthosis, with pseudoepitheliomatous hyperplasia, resulting in long rete pegs. Intraepithelial microabscesses were common. Several multinucleated giant cells were often observed, frequently organized in granulomas interspersed by severe chronic inflammation composed mainly by lymphocytes and macrophages. In all cases, *P. brasiliensis* was found mainly in the connective tissue, either inside of multinucleated giant cells or extracellularly (Fig. 2). Gomori-Grocott staining confirmed the presence of yeasts, which appeared as globous aspect impregnated by silver. Some of them presented multiple buddings with mickey ear appearance (Fig. 2).

## Discussion

Between 1930 and 2012, more than 15,000 cases of PCM have been reported in Latin America. Among the countries in this region, Brazil shows the highest prevalence with 12,476 cases, followed by Colombia (940 cases), and Venezuela (674 cases) (3). Particularly in Brazil, the South, Southeast and Midwest are considered endemic areas of PCM. On the other hand, most of North and Northeastern Brazil is a non-endemic region, including the Pernambuco state (3,8). In Brazil, the mortality rate of PCM is 1.45 cases per million inhabitants, making it the eighth leading cause of death by infectious chronic diseases (9). In addition, PCM is an important cause of morbidity, mainly in those cases with spread disease, including the oral cavity. Thus, knowing the clinical features of oral PCM is essential to establish a prompt diagnosis and appropriate treatment. This can be challenging in non-endemic areas, such as in the region evaluated in this study.

In this study, five of the six cases reported were of patients living in Northeastern Brazil. Only one patient lived in Sao Paulo state, an area in Brazil where PCM is endemic, and none of the other 5 patients ever visited an endemic region. Although the Brazilian Northeast has low endemicity (3), and is the region with the lowest mortality rates for PCM, this Brazilian region has high death rates and high percentages of deaths due to ill-defined causes. Therefore, the low mortality rate of PCM may be due to the high percentage of deaths classified as having unknown causes, in addition to the limited availability of health services and case resolution (9). Moreover, the low endemicity in this region may also contribute for misdiagnosis of PCM.

PCM can occur in any age group, but there is a higher prevalence between the fourth and seventh decades of life. In our study, the mean age of patients affected by PCM was 53.8 years, which is similar to results of other researches (3,10). The PCM has a large preference for males (3,7,8,10), as observed in our study, with all patients being male. Women are less often affected because of the female hormone estradiol 17-β, which inhibits the transition of mycelium or conidium to yeast (pathogenic form), preventing the progression of the disease (11,12).

The relationship between PCM and rural activities is well established in literature (12,13). Moreover, the most common occupation of the patients was that of current or retired rural worker. This association is plausible since activities related to soil management, such as agricultural activities, earthworks, soil preparation, gardening practices, and transportation of plant products, involve direct contact with the habitat of P. brasiliensis. This emphasizes the importance of taking the occupation history of the patients properly, to assistance in the differential diagnosis of the lesions, as well as to document important epidemiological data. In addition, patients who smoke and drink alcohol present higher risk of PCM (14). Although the association between PCM and immunosuppressive diseases is not as common as in other systemic mycoses, there are reports of PCM in transplanted patients with malignant neoplasms or living with HIV/AIDS (2,15).

The most common signs and symptoms of PCM in the chronic form are lung, larynx, and integument involvement, represented by cough, dyspnea, mucopurulent sputum, and cutaneous ulcers. Pharyngeal ulcers result in odynophagia, dysphagia, and dysphonia. Systemic signs include fatigue, weight loss, lower blood pressure, and skin darkening. In addition, lymphadenopathy, abdominal pain, headache, motor deficit, and behavior change and/or level of consciousness may occur (12).

Oral manifestations are quite frequent and often represent the first clinical sign of the disease, usually occurring in all cases of PCM (8,16). The oral lesions of PCM usually appear as multiple painful and moriform ulcers (9,12,13), mainly affecting the buccal and labial mucosa, gingiva, and palate (5,8,10), similar to those found in this study. However, the oral manifestation may be a single ulcer (17). Overall, the studies showed long development time of the oral manifestations (8,10,16), which may be attributed to delay in seeking a health service or the difficulty of practitioners to establish the diagnosis of PCM.

In the present study, squamous cell carcinoma was the most considered differential diagnosis, followed by PCM. Additionally, leishmaniasis, histoplasmosis, and actinic cheilitis were considered in one case. Squamous cell carcinomas, leishmaniasis, cryptococcosis, histoplasmosis, aspergillosis, syphilis, tuberculosis, and granulomatosis with polyangiitis are considered in the differential diagnosis of oral PCM (12,18). The oral squamous cell carcinoma is the main differential diagnosis of oral PCM (12,18), which is compatible with the findings in our study. The clinical diagnosis may be challenging in cases with single oral lesions (17) or when occurring in non-endemic areas. In this study, only in the case 4, which was from an endemic region, PCM was the only disease considered in the differential diagnosis. In 2 cases (2 and 3), PCM was not raised.

Incisional biopsy of oral lesions is indicated for subsequent histopathological analysis to determine the diagnosis. Hematoxylin-eosin, PAS, and Grocott-Gomori stain were the stains used in histopathological examination. The connective tissue showed severe chronic inflammatory reaction and granuloma formation with multinucleated giant cells. Yeasts were found inside the multinucleated giant cells or extracellularly. Microscopically, oral PCM may also mimic a squamous cell carcinoma. Pseudoepitheliomatous hyperplasia that occurs in oral PCM has several characteristics similar to a well-differentiated squamous cell carcinoma (18). However, a PCM does not have the tendency to keratinization. Moreover, the pseudoepitheliomatous hyperplasia shows intense intraepithelial inflammatory infiltration, usually with microabscess formation (19). The establishment of PCM diagnosis depends on the identification of P. brasiliensis. The fungus is more easily observed through special stains such as Grocott-Gomori and PAS, where the fungus often appears as mickey ear or rudder (20). Additionally, the diagnosis can be made through exfoliative cytology examination, isolation, culture of the microorganism, and serological techniques (15).

Unlike other fungi, *P. brasiliensis* is sensitive to most antifungals. Thus, for the treatment of PCM, fluconazole, ketoconazole, or itraconazole may be used, which are drugs that cause disturbance in the fungal membrane permeability by inhibiting the synthesis of ergosterol. For severe PCM, amphotericin B is indicated. However, the amphotericin B is a potentially nephrotoxic drug. Hence, the medical professional must be attentive to the systemic health condition of the patient before prescribing it (21). In this study, as the cases studied were from an oral pathology laboratory, the clinical information on treatment of the patients as well other exams, were not available.

## Conclusions

In conclusion, PCM is rare in the Brazilian northeastern region. Despite of this, this deep mycosis present epidemiological and clinical features similar those observed in endemic regions from Brazil, affecting mainly adults between 30 and 60 years of age, men, those involved in rural activities, and residents in rural areas. Oral manifestations include multiple, ulcerated, erythematous, or granular lesions affecting preferentially the buccal mucosa, soft and hard palate, and gingiva. Thus, the practitioners should be aware of the clinical characteristics of PCM, since oral manifestations are often the first clinical sign of the disease. The PCM should be considered in the differential diagnosis of chronic oral ulcers, whether they are single or especially those that develop multiple.
